# Hyperkalemic Periodic Paralysis Secondary to End-Stage Renal Disease and Excess Potato Consumption

**DOI:** 10.7759/cureus.57321

**Published:** 2024-03-31

**Authors:** Amy E Hunt, Adam Crawford, Gretchen Newman, Scott A Area

**Affiliations:** 1 Emergency Medicine, Charleston Area Medical Center, Charleston, USA

**Keywords:** diet modification, stroke-like symptoms, severe hyperkalemia, ascending flaccid paralysis, end-stage renal disease (esrd), cardiac dysrhythmia, hyperkalemic periodic paralysis

## Abstract

Hyperkalemic periodic paralysis is a rare medical condition characterized by periods of extreme muscle weakness or paralysis. While most cases of hyperkalemic periodic paralysis are associated with a genetic channelopathy, cases of secondary hyperkalemic periodic paralysis can pose a challenge for medical personnel in terms of timely recognition. Identification of this medical emergency early in its course is essential to preventing cardiac and neurological sequelae. We report a case of a 58-year-old female who presented with stroke-like symptoms and was found to have secondary hyperkalemic periodic paralysis attributed to the excess consumption of potatoes, a potassium-rich food. This case highlights the importance of considering hyperkalemic periodic paralysis early in the differentiation of patients with end-stage renal disease (ESRD) who present with muscle weakness and stroke-like symptoms.

## Introduction

Hyperkalemia is a potentially life-threatening electrolyte abnormality that can affect cardiac conduction and cardiac contractility and ultimately lead to cardiac arrest [1]. According to Mishra et al., hyperkalemic periodic paralysis is a unique and uncommon presentation of hyperkalemia that is characterized by flaccid motor weakness. If this electrolyte imbalance is not recognized early, it can be life-threatening for patients. Hyperkalemic periodic paralysis can be acquired; it is commonly inherited genetically in the form of channelopathy. Secondary hyperkalemic periodic paralysis is seen less frequently and is attributed secondary to other disease processes or etiologies patients may be unaware of [2]. Obtaining a thorough medical history and recognizing electrocardiogram (ECG) findings of hyperkalemia are essential to identifying this neurologic manifestation so that treatment is not delayed. The authors present a patient with hyperkalemic periodic paralysis secondary to a potassium-rich diet whose symptoms rapidly resolved due to the clinician’s astute recognition of ECG findings and their ability to obtain a thorough medical history. 

## Case presentation

A 58-year-old Caucasian female with a history of end-stage renal disease (ESRD) on hemodialysis presented to the emergency department (ED) via emergency medical services (EMS) for concern of an acute stroke. The patient stated that 18 hours prior to arrival, she was unable to move her lower extremities. Her symptoms had progressed rapidly to the point that she was now unable to move her upper extremities. The patient informed the ED physicians that she dialyzed to completion two days prior and had not missed any of her scheduled hemodialysis appointments. Further investigation into the patient’s history revealed that since her last dialysis session, she had been consuming a diet comprised of mostly potatoes but was unable to provide further details about her diet. In addition to ESRD on hemodialysis, the patient’s medical history included hypertension, diabetes mellitus, hyperlipidemia, and chronic obstructive pulmonary disease (COPD). Notable home medications included a 90-mcg ProAir inhaler, a 160/4.5-mcg Symbicort inhaler, an insulin aspart sliding scale before meals, and 20 units of insulin glargine every other day. She denied taking any of these home medications the day before when her symptoms started. The patient also denied trauma, recent vaccinations, infections, or taking any diuretics or medications that could increase her potassium level. Family history was non-contributory, and social history was significant only for tobacco abuse. 

While in the ED, the patient’s vital signs demonstrated a heart rate of 49 beats per minute, a temperature of 37.9 °C, a blood pressure of 119/65 mmHg, an oxygen saturation of 100% on room air, and a respiratory rate of 20 breaths per minute. The patient’s physical examination revealed an alert, keenly responsive female who could not move her bilateral upper and lower extremities. She did, however, have preserved sensation across all four extremities. The patient did not have facial asymmetry, aphasia, dysarthria, or hemispatial neglect, and visual field testing was normal. Her National Institute of Health Stroke Scale (NIHSS) score was eight, indicating moderate severity of stroke symptoms.

**Figure 1:**
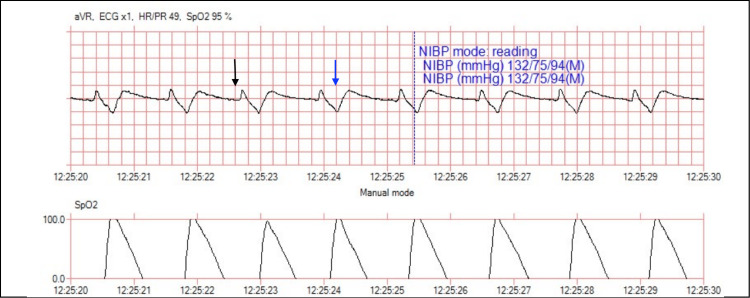
An electrocardiogram performed by the emergency medical services paramedics at 12:25 hours demonstrated the loss of P wave morphology (black arrow) and the broadening of the QRS complex (blue arrow).

A rhythm strip completed by EMS immediately upon the patient’s arrival at the ED showed bradycardia with a heart rate of 49 beats per minute and a wide QRS complex (Figure 1). Upon identification of this life-threatening rhythm, the patient was immediately administered 2 g of intravenous (IV) calcium gluconate and 50 mEq IV sodium bicarbonate while awaiting the return of laboratory tests. Due to her acute neurologic symptoms, the patient then expeditiously received a head computed tomography (CT) scan, which was negative for ischemia or intracerebral hemorrhage. Upon completion of the head CT, a laboratory workup was obtained, and the patient’s chemistry panel revealed a sodium level of 120 mmol/L, potassium of 8.5 mmol/L, chloride of 94 mmol/L, creatinine of 5.0 mg/dL, blood urea nitrogen of 43 mg/dL, and glucose level of 126 mg/dL. The complete blood count (CBC) was within normal limits.

**Figure 2:**
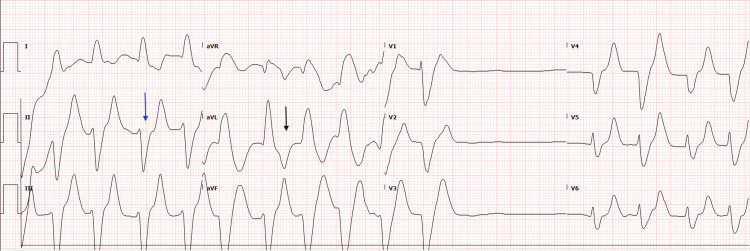
The initial electrocardiogram performed in the emergency department at 13:03 hours showed marked intraventricular delay with a prolonged QRS at 216 ms (blue arrow). QTc was also prolonged at 617 ms (black arrow).

**Figure 3:**
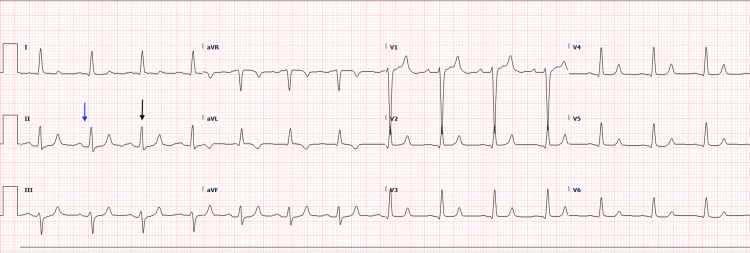
An electrocardiogram performed at 13:26 hours after the administration of potassium-lowering medications showed a normal sinus rhythm with a heart rate of 86 beats per minute. The PR interval improved to 198 ms (blue arrow), and the QRS interval improved to 103 ms (black arrow).

An initial 12-lead ECG performed in the ED showed a wide complex tachycardia with intraventricular conduction delay, a heart rate of 98 beats per minute, and a widened and prolonged QRS interval at 216 ms (Figure 2). After review of the ECG and obtaining a critical potassium level of 8.5 mmol/L, the patient was administered an additional 1g IV calcium gluconate, 150 mEq IV sodium bicarbonate, 10 units of IV regular insulin, 25 g IV dextrose, 10 g of oral sodium-zirconium cyclosilicate, and a 90 mcg albuterol breathing treatment. Within 20 minutes of receiving these medications, the patient was able to move all four extremities and displayed improved motor strength. A repeat ECG was obtained 23 minutes after the patient’s initial ECG and demonstrated a conversion to normal sinus rhythm with an improved QRS interval of 103 ms (Figure 3). The patient’s potassium level also improved to 7.6 mmol/L approximately 30 minutes after receiving the potassium-lowering medications.

After stabilization in the ED, the patient was admitted to the intensive care unit (ICU) for further care, including emergent hemodialysis. Post-dialysis, the patient’s serum potassium and sodium levels normalized to 3.9 mmol/L and 131 mmol/L, respectively. Considering the patient’s historical sodium baseline was 130 mmol/L, the consulting nephrologist believed the initial whole blood sodium level of 120 mmol/L was an erroneous result. The remainder of the patient’s stay was uneventful, and she was discharged after three days of hospitalization.

## Discussion

Hyperkalemia, defined as a serum potassium level greater than 6 mmol/L, is a potentially life-threatening electrolyte abnormality seen in patients secondary to either dietary intake or impaired excretion of potassium [3]. Patients with severe hyperkalemia can have life-threatening symptoms such as cardiac dysrhythmias and conduction abnormalities, as well as muscle weakness that can progress to paralysis [4]. The paralysis associated with hyperkalemia starts distally in the lower extremities and ascends to involve the patient’s trunk and upper extremities with little to no sensory impairment [5]. Muscle paralysis associated with a hyperkalemic state has been coined "hyperkalemic periodic paralysis". Hyperkalemic periodic paralysis has been described in two forms: primary hyperkalemic periodic paralysis and secondary hyperkalemic periodic paralysis. Primary hyperkalemic paralysis (hyperkalemic periodic paralysis) is an inherited autosomal dominant disorder that impairs sodium channel function in skeletal muscle cells and thereby reduces the ability to regulate potassium levels [2]. Secondary hyperkalemic periodic paralysis is the acquired form of the disorder. With secondary hyperkalemic periodic paralysis, neurophysiological testing has revealed that although there is normal muscle fiber activity, there is an interruption in nerve conduction rate within the skeletal muscle. Secondary hyperkalemic periodic paralysis is most commonly seen in patients with acute or chronic renal dysfunction, Addison’s disease, rhabdomyolysis, or excessive ingestion of potassium supplements or potassium-rich foods such as bananas or potatoes [2]. Our patient, unfortunately, had not received any dietary education since her diagnosis of ESRD six months prior and, as a result, continued to ingest large quantities of potatoes. 

With hyperkalemia as a potential cause of paralysis and cardiac arrhythmias, it is imperative to quickly initiate treatment to lower the potassium level. Variations in ECG patterns are known to be directly correlated with serum potassium levels, but classic ECG findings suggesting hyperkalemia, such as tall, peaked T waves, prolonged PR interval, or widening of the QRS, are rare and only occur in 45.45% of patients [5, 6]. Furthermore, uremic patients with ESRD have even fewer significant ECG changes consistent with hyperkalemia [7]. Such a presentation was reported by Bangolo et al., which described a case of a patient with a potassium level of 9.6 mmol/L who had ascending paralysis but no ECG findings consistent with hyperkalemia [3]. With this in mind, it is difficult, at times, to discern life-threatening hyperkalemia based on ECG findings, and treatment for the electrolyte derangement is delayed. Our case is unique in that the patient’s ECG findings did suggest severe hyperkalemia. The astute recognition of the amplified R wave, widened QRS, and bradycardia on the initial rhythm strip from EMS allowed the ED physicians the opportunity to treat the potential hyperkalemia before a 12-lead ECG and laboratory values were obtained. 

Reported cases of concomitant paralysis with ECG findings suggestive of hyperkalemia are even rarer, highlighting the uniqueness of this patient’s presentation. In our case, a patient developed severe hyperkalemia secondary to excessive consumption of potatoes, a potassium-rich food, in the setting of chronic renal insufficiency. As a result, she experienced stroke-like symptoms with acute paralysis of all four extremities. There have been just three case reports in the literature to date where patients who presented with hemiparesis were found to have severe hyperkalemia. Specifically, Bahrami et al. illustrate a patient with the acute onset of stroke-like symptoms of hemiparesis and slurred speech. Due to concern for an acute ischemic stroke, the patient received an intravenous recombinant tissue plasminogen activator (IV-rTPA); the patient did not experience any improvement in their stroke-like symptoms. Concomitantly, the patient was also found to have a potassium level of 9 mEq/L with EKG changes, and upon receiving treatment for the hyperkalemia, the patient’s neurologic deficits improved [8]. Our case is unique in that providers were able to initiate expeditious treatment for hyperkalemia based on the recognition of an ECG finding before proceeding with a stroke evaluation. As a result, the patient’s stroke symptoms resolved within one hour of arriving at the ED. Our case highlights that in patients presenting with stroke-like symptoms with ECG changes, hyperkalemic periodic paralysis should be considered within the differential of their neurologic deficits.

## Conclusions

Hyperkalemic periodic paralysis is a rare, neurologic manifestation of severe hyperkalemia. Our case exemplified the importance of considering hyperkalemic periodic paralysis in ESRD patients who present with motor impairment and other stroke-like symptoms. Recognizing this condition early in a patient’s course can expedite treatment and, as our case has shown, rapidly improve the patient's symptoms. Furthermore, this case shows the importance of patient education and the impact it may have on patient health.
